# Unusual radiological presentation of extralobar pulmonary sequestration: a preoperative diagnostic challenge

**DOI:** 10.1186/s44215-026-00258-1

**Published:** 2026-04-22

**Authors:** Yuya Nobori, Takehiro Tsuchiya, Masaki Anraku

**Affiliations:** Department of Thoracic Surgery, Tokyo Metropolitan Institute for Geriatrics and Gerontology, 35-2 Sakaecho, Itabashi-ku, Tokyo, 173-0015 Japan

**Keywords:** Extralobar pulmonary sequestration, Absence of the left pericardium

## Abstract

**Background:**

Extralobar pulmonary sequestration is a rare disease and is frequently associated with congenital malformations. Herein, we report an adult case of an unusual extralobar sequestration accompanied by a bronchogenic cyst and the concurrent absence of the left pericardium.

**Case presentation:**

A 57-year-old female patient presented with an abnormal shadow on a chest radiograph. Contrast-enhanced computed tomography revealed a partially enhanced cystic mass in the left anterior mediastinum. Another cystic lesion was detected on the left side of the trachea in the upper mediastinum. The anterior mediastinal mass was suspected to be a cystic thymoma, and thus, a left thoracoscopic surgery was performed for its resection. The mass originated from a left pulmonary hilar region and was covered by an independent visceral pleura. The feeding artery originated from the left pulmonary artery with no connection to the bronchus. Histopathological results revealed that the mass consisted of alveolar cells, bronchial epithelium, cartilage, and a cystic structure with highly viscous contents, and it was finally diagnosed as extralobar sequestration.

**Conclusions:**

Extralobar sequestration should be considered a differential diagnosis for atypical anterior mediastinal masses near the hilar region, especially in patients with other congenital malformations.

**Supplementary Information:**

The online version contains supplementary material available at 10.1186/s44215-026-00258-1.

## Background

Pulmonary sequestration, a rare congenital thoracic anomaly, is characterized by nonfunctioning lung parenchyma and pleura that receive systemic arterial blood supply and lack communication with the tracheobronchial tree, resulting in impaired ventilation. It is classified into intralobar sequestration, which coexists with normal lung tissue, and extralobar sequestration (ELS), which exists independently. ELS is more commonly diagnosed in males and pediatric patients and typically arises between the left lower lobe and diaphragm, with blood supply from the aorta [1]. It is frequently associated with congenital anomalies, including diaphragmatic hernia and cardiac abnormalities. Rare adult cases with pulmonary arterial supply and unusual mediastinal locations present diagnostic challenges. Herein, we report a rare case of an adult female with anterior hilar ELS.

### Case presentation

**Fig. 1:**
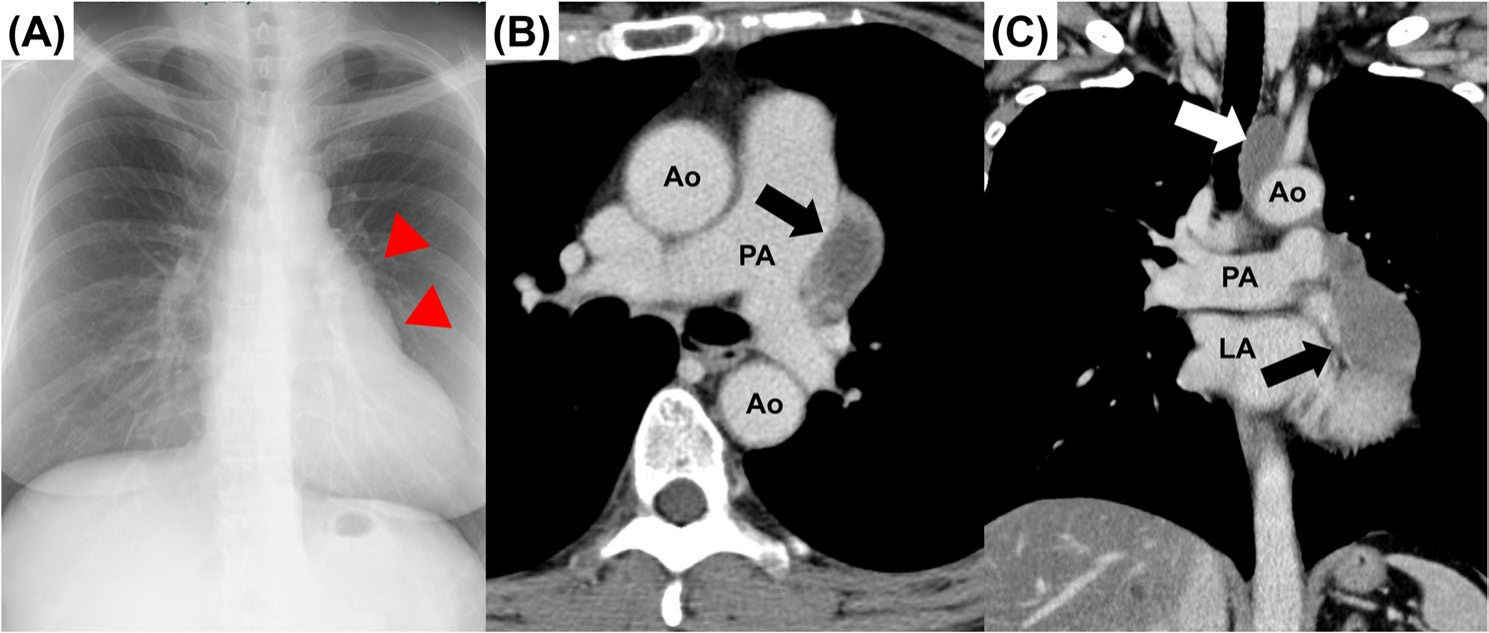
Radiological findings. (**A**) Abnormal shadow at the left hilum on chest radiograph (red triangle). (**B**) Horizontal computed tomography image showing a partially encapsulated mass adjacent to the left pulmonary artery (black arrow). (**C**) Coronal computed tomography image showing a mass at the left hilum (black arrow) and a cyst on the left side of the trachea (white arrow). Ao: aorta; PA: pulmonary artery; LA: left atrium

A 57-year-old female patient presented with an abnormal shadow on an annual screening chest X-ray (Fig. 1A). She was asymptomatic with no medical history, and physical examination was unremarkable. Contrast-enhanced computed tomography (CT) revealed a partially enhanced cystic mass in the anterior mediastinum with a maximum diameter of 60 mm (Fig. 1B–C). Magnetic resonance imaging (MRI) confirmed predominantly cystic components. Positron emission tomography showed minimal abnormal uptake. Tumor markers and anti-acetylcholine receptor (AChR) antibody levels were within normal ranges. Echocardiography revealed irregular cardiac motion in the left lateral recumbent position, suggesting a left pericardial defect. The anterior mediastinal mass was suspected to be a cystic thymoma. Additionally, another cystic mass was identified on the left side of the trachea in the upper mediastinum, suspected to be a bronchogenic cyst.

**Fig. 2:**
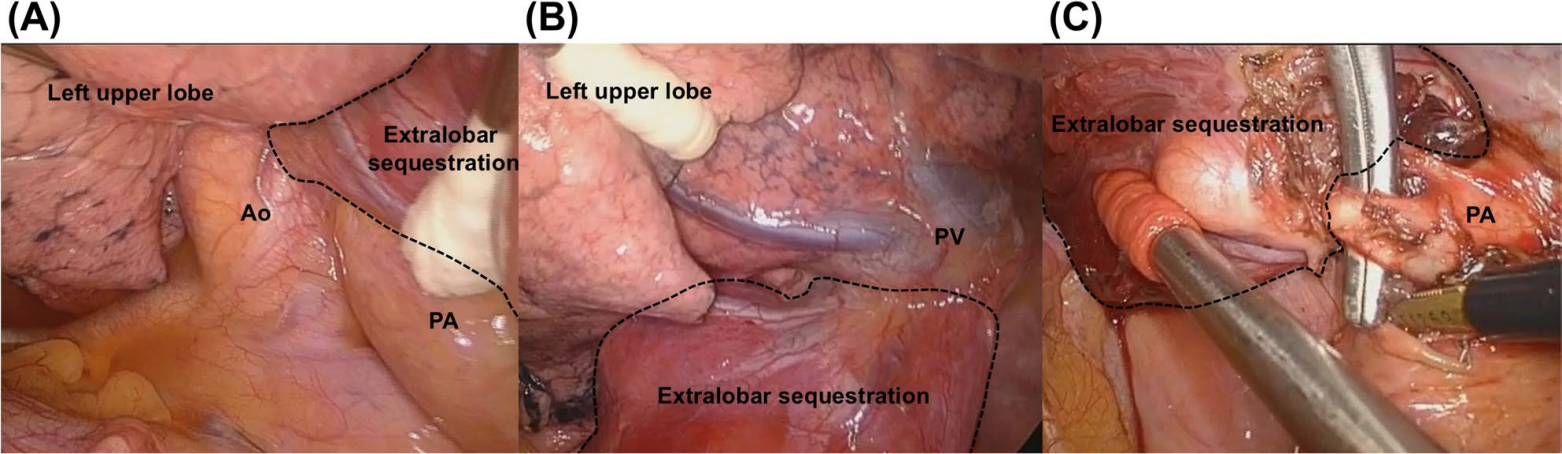
Intraoperative findings. (**A**) The extralobar pulmonary sequestration was located ventral to the left upper lobe, arising caudal to the aorta. (**B**) The sequestration drained into the left superior pulmonary vein. (**C**) The feeding artery to the sequestration originated from the proximal left pulmonary artery. Ao: Aorta; PA: pulmonary artery; PV: pulmonary vein

Left thoracoscopic surgery was performed to resect the anterior mediastinal mass (see Additional file 1). The mass originated from the left pulmonary hilum and was located independently outside the mediastinum and left lung lobes, covered by visceral pleura and separate from normal lung tissue (Fig. 2A–B). The left pericardium was completely absent, exposing the heart to the left pleural cavity. The mass was attached to the main pulmonary artery and superior pulmonary vein, which were dissected using surgical staplers (Fig. 2C). It showed no connection to the left bronchus and was resected after dissection of the left hilum. Another upper mediastinal cyst was found on the left side of the trachea, and its cyst wall was excised to drain blood clot–like contents. The thoracoscopic surgery lasted 191 min, with a blood loss of 53 mL.

**Fig. 3:**
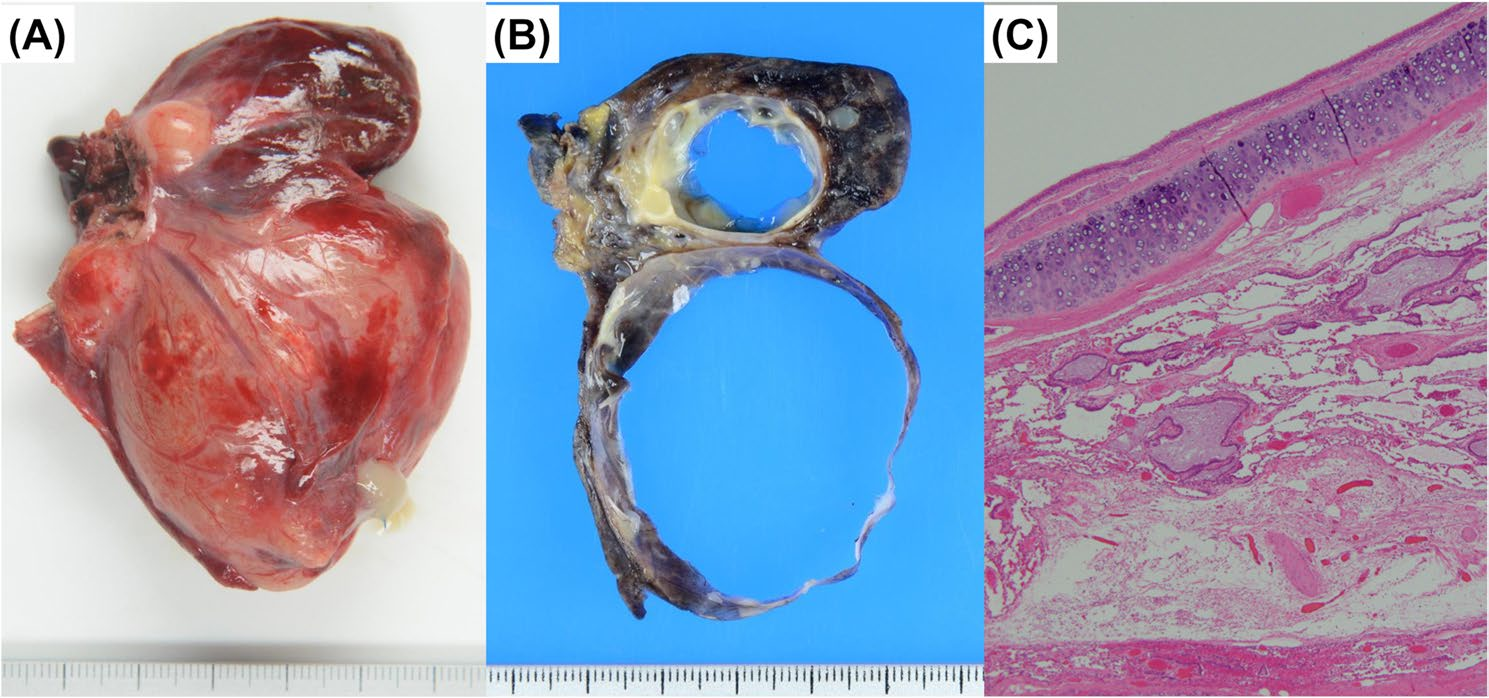
Pathological findings. (**A**) Gross appearance of the resected mass in the hilum. (**B**) Section of the mass in the left hilum after formalin fixation. (**C**) Histopathological findings showing lung alveoli, bronchial mucosa, and cartilage

Histopathological examination revealed that the mass was covered by visceral pleura and contained lung parenchyma (Fig. 3A–C). The cystic lesion with highly viscous contents was composed of bronchial epithelium surrounded by cartilage and glands. No malignancy was identified, and the mass was diagnosed as extralobar pulmonary sequestration. The upper mediastinal mass wall showed bronchial epithelium, consistent with a bronchogenic cyst.

## Discussion

Diagnosis of extralobar pulmonary sequestration in a woman in her 50s, particularly when accompanied by congenital anomalies, is exceedingly rare. Pulmonary sequestration accounts for only 0.15%–6.4% of all congenital pulmonary malformations [1], and only four cases with complete absence of the left pericardium have been reported, all diagnosed in individuals aged 20–39 years [2–5] (Table 1). In contrast, the present case was diagnosed at 57 years, beyond the previously recognized age range. It is possible that the abnormal shadow near the left hilum on chest radiography had been overlooked for a long time.

**Table 1:** Reported cases of extralobar pulmonary sequestration associated with absence of the pericardium (*: present case)

Case	Age	Sex	Side	Location	Feeding vessel	Associated abnormalities	Preoperative diagnosis	Reference
1	35	M	Left	Anterior mediastinum	Pulmonary artery	Bronchogenic cyst	Congenital abnormality	[2]
2	32	M	Left	Anterior mediastinum	Pulmonary artery	None	Thymoma	[3]
3	22	M	Left	Anterior mediastinum	Pulmonary artery	Bronchogenic cyst	Thymoma	[4]
4	22	F	Left	Anterior mediastinum	Pulmonary artery	Atrial septal defect	Cystic teratoma	[5]
*	57	F	Left	Anterior mediastinum	Pulmonary artery	Bronchogenic cyst	Cystic thymoma	Present case

This case highlights a preoperative diagnostic challenge, as a definitive diagnosis could not be established before surgery. Pulmonary sequestration was not suspected because the lesion appeared radiographically as an anterior mediastinal mass without identifiable systemic arterial supply. In addition, the feeding vessel originated from the pulmonary artery, which is atypical for sequestration and further obscured the diagnosis. Notably, all previously reported cases were also not diagnosed preoperatively, underscoring the difficulty of accurate preoperative diagnosis in this condition (Table 1). Interestingly, all reported cases of extralobar sequestration located in the anterior mediastinum, including the present case, were supplied by the pulmonary artery rather than the systemic circulation. Pulmonary arterial supply is uncommon and has been reported only rarely, particularly in extralobar sequestration, with only a limited number of cases described in the literature. Although the differential diagnosis initially favored an anterior mediastinal tumor, the possibility of an intrathoracic tumor should have been considered, given the lesion’s proximity to the left pulmonary hilum and the presence of bronchogenic cysts. Furthermore, suspicion of a left pericardial defect on echocardiography should have prompted consideration of rare congenital conditions, including ELS.

Surgical resection is recommended in most ELS cases to disconnect abnormal vessels and remove sequestered lung tissue. In this case, given the patient’s age of 57 years, concern for malignancy justified surgical resection. Although preoperative identification of vascular anatomy is desirable for safe surgery, neither the diagnosis nor the feeding artery could be identified preoperatively. Retrospective review suggested that the feeding vessel was not clearly visualized on contrast-enhanced CT, possibly because of limitations in spatial resolution and the lack of optimized arterial-phase imaging. A multiphase contrast-enhanced study, including arterial and venous phases, might have improved detection of the vascular connection. Careful intraoperative identification and ligation of feeding vessels are essential to avoid massive hemorrhage because the feeding artery originated from the main pulmonary artery trunk, proximal to the segmental pulmonary artery branch (A3). Thoracoscopic surgery was successfully performed in this case. Pericardial reconstruction was not performed because the left lung was preserved and no intraoperative evidence of cardiac instability was observed. Although the risk of cardiac herniation was considered low in this setting, careful postoperative observation was required. In previously reported cases of ELS with left pericardial defect, reconstruction was not always performed.

## Supplementary Information

Additional file 1. Title: Video of thoracoscopic surgery for extralobar sequestration. The left anterior mass originated from left hilar region. The left pericardium was completely absent. The mass was resected after dissecting the vessels that communicate with the pulmonary artery (PA) and pulmonary vein (PV). Ao: Aorta; PA: pulmonary artery; PV: pulmonary vein.

## Data Availability

No data were generated or analysed.

## Abbreviations

ELS: Extralobar sequestration
